# Effect of Tirzepatide in an Adolescent With Early-Onset Obesity, Hyperphagia, and Type 2 Diabetes

**DOI:** 10.7759/cureus.94770

**Published:** 2025-10-17

**Authors:** Bakht Noor Khurshid, Ukasha Moazzam, Ioannis Dimitropoulos

**Affiliations:** 1 Acute Medicine, University Hospitals Plymouth NHS Trust, Plymouth, GBR; 2 Geriatrics, University Hospitals Plymouth NHS Trust, Plymouth, GBR; 3 Diabetes, Endocrinology and General Internal Medicine, University Hospitals Plymouth NHS Trust, Plymouth, GBR

**Keywords:** adolescent hyperphagia, dual incretin therapy, early-onset obesity, hypothalamic obesity, neurodevelopmental disorders, tirzepatide therapy, type 2 diabetes mellitus

## Abstract

Early-onset severe obesity is rare and largely treatment-resistant to standard measures. Etiologies include syndromic disorders (Prader-Willi, Bardet-Biedl and Alström syndromes), monogenic defects of appetite-regulating pathways - most significantly melanocortin-4-receptor (MC4R) deficiency, but also leptin receptor (LEPR), pro-opiomelanocortin (POMC), and secondary hypothalamic injury, most commonly craniopharyngiomas. Most childhood obesity, however, is polygenic, arising from interactions of environmental and genetic factors. In many patients, no clear etiology is identified. Treatment remains challenging, particularly if hyperphagia and reduced satiety are predominant presenting features.

We describe the case of a 19-year-old male with a history of autism spectrum disorder, attention-deficit hyperactivity disorder (ADHD), type 2 diabetes mellitus (T2DM) and morbid obesity, who showed a clinically significant response to tirzepatide therapy following failed conventional interventions.

This case highlights the potential role of dual incretin therapy in adolescents with severe early-onset obesity and hyperphagia, even in the absence (based on present knowledge) of identifiable hypothalamic or genetic pathology.

## Introduction

Childhood obesity is defined as BMI ≥95th percentile for age and sex; severe obesity as ≥120% of the 95th percentile or ≥35 kg/m², whichever is lower [1]. The American Academy of Pediatrics (AAP) clinical practice guideline (2023) further categorises severe obesity into Class 2 (≥120% to <140% of the 95th percentile or BMI ≥35 to <40 kg/m², whichever is lower) and Class 3 (≥140% of the 95th percentile or BMI ≥40 kg/m², whichever is lower) [2]. These thresholds are widely used in clinical and research contexts, including genetic-obesity reviews [3]. Severe obesity beginning in childhood or adolescence is uncommon and, particularly when accompanied by hyperphagia, should prompt evaluation for syndromic or monogenic aetiologies [1-3].

Recognised causes of early-onset obesity include syndromic disorders such as Prader-Willi, Bardet-Biedl and Alström syndromes, monogenic mutations affecting the leptin-melanocortin pathway (including LEP, LEPR, pro-opiomelanocortin (POMC), melanocortin-4-receptor (MC4R)), and secondary hypothalamic damage following tumours, trauma, or irradiation [3]. In many cases, however, no structural, syndromic or monogenic explanation is found. In such patients, hyperphagia may reflect disturbances in homeostatic mechanisms (mediated by hypothalamic integration of peripheral signals such as leptin, insulin and glucagon-like peptide-1 [GLP-1]) and hedonic pathways (dopaminergic reward circuits that drive food-seeking behaviour) [4,5].

Therapeutic options for severe early-onset obesity remain limited, with conventional dietary and pharmacological interventions frequently proving inadequate. GLP-1 receptor agonists such as liraglutide and semaglutide have recently demonstrated benefit in adolescents with obesity [2]. Tirzepatide, a dual agonist at GLP-1 and glucose-dependent insulinotropic polypeptide (GIP) receptors, extends this pharmacological approach and has demonstrated robust weight loss and glycaemic benefits in adults with type 2 diabetes and obesity [6]. Its mechanisms include augmentation of satiety signalling, suppression of hedonic food drive, improved insulin sensitivity, and reduced gastric emptying. The potential of tirzepatide in young patients with refractory hyperphagia and obesity is only beginning to be explored [7-9].

We present a case of an adolescent male with severe early-onset obesity, hyperphagia, and type 2 diabetes, without identifiable syndromic or monogenic cause, who experienced marked clinical improvement with tirzepatide therapy.

## Case presentation

A 19-year-old male with a history of autism spectrum disorder, attention-deficit hyperactivity disorder (ADHD), lifelong obesity from early childhood, morbid obesity (BMI 54.3 kg/m²), type 2 diabetes mellitus, non-alcoholic fatty liver disease, and a recent diagnosis of chronic pulmonary embolism, presented with severe hyperphagia, impaired satiety, and progressive weight gain since early adolescence. His diabetes was poorly controlled on metformin modified-release 1 g twice daily, with an initial hemoglobin A1c (HbA1c) of 96 mmol/mol.

He also underwent L2-L4 intersegmental decompressions and discectomies in July 2025 due to worsening back pain. There was no history of hypothalamic tumour, cranial irradiation, or neurosurgical intervention.

He was enrolled in the Genetics of Obesity Study (GOOS), where monogenic obesity testing, including MC4R mutation screening, was negative. There was normal growth and development in childhood with normal secondary sexual characteristics, including normal body and facial hair distribution, and absence of gynaecomastia. These findings made syndromic causes of obesity, such as Prader-Willi and Bardet-Biedl, unlikely.

Thyroid function tests were normal, which successfully ruled out hypothyroidism as an etiology. Biochemistry showed mild secondary hypogonadism alone. In addition, an MRI of the brain performed in March 2017 demonstrated normal hypothalamus and pituitary.

Autoimmune diabetes was virtually excluded with negative glutamic acid decarboxylase (GAD), insulinoma-associated antigen-2 (IA-2), and zinc transporter 8 (ZnT8) antibodies.

**Figure 1:**
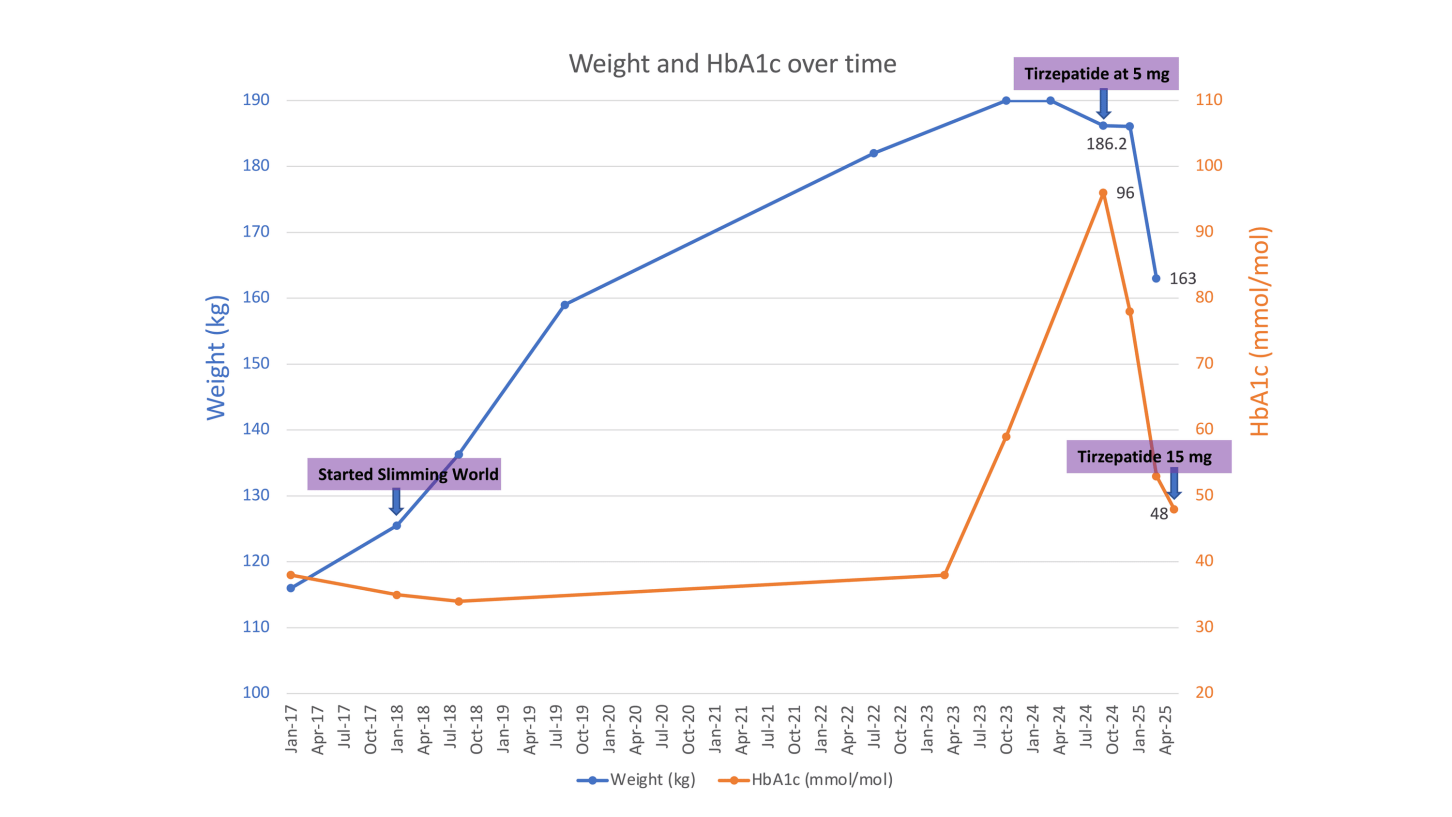
The graph illustrates the trajectory of hemoglobin A1c (HbA1c) and body weight over time, as related to dietary intervention and tirzepatide dose titration.

Initial pharmacological management was a trial of lisdexamfetamine (Elvanse), an appetite suppressant used in ADHD, with no effect on satiety or food intake. Another medication, guanfacine, was also considered but not initiated. The patient had already attempted a number of structured lifestyle interventions in the past, including a Slimming World programme, with minimal success. Given the persistent weight gain, voracious appetite, suboptimal glycaemic control, and emerging secondary complications of obesity, tirzepatide was initiated at 2.5 mg once weekly and subsequently up-titrated to 5 mg. This resulted in a profound reduction in appetite with increased satiety, and HbA1c decline from 96 to 78 mmol/mol and weight loss from 186 to 174 kg within four months. Subsequent dose titration to 15 mg weekly was followed by further weight loss to 163 kg and HbA1c reduction to 48 mmol/mol by May 2025 (Figure 1). Adverse effects were limited to transient post-injection nausea and a brief, self-limited diarrhoea the following day; both resolved without treatment.

**Figure 2:**
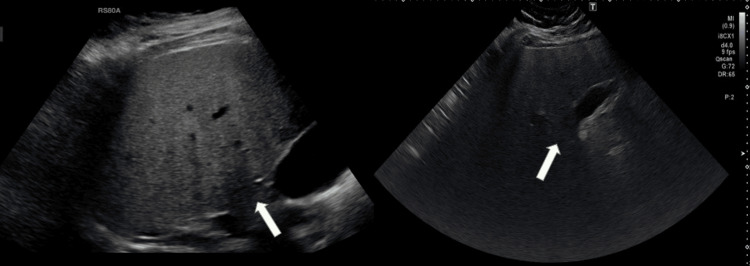
US Abdomen (2015, left; 2024, right) showing hepatic steatosis. White arrows indicate hepatic parenchyma with increased echogenicity and posterior beam attenuation in 2024 compared to 2015.

Previous abdominal ultrasound (2015) demonstrated severe hepatic steatosis with borderline splenomegaly. Repeat ultrasound in 2024 confirmed progression (Figure 2). Transient elastography (FibroScan) in May 2025 revealed a stiffness of 11.3 kPa, consistent with severe fibrosis. Notably, alanine aminotransferase (ALT) improved from 99 to 35 U/L over the preceding year, reflecting decreased hepatic inflammation.

## Discussion

Severe obesity with early onset is uncommon and is often refractory to lifestyle or standard pharmacologic treatment; when accompanied by hyperphagia and impaired satiety, it should prompt evaluation for syndromic or monogenic etiologies. This is explicitly recommended by the Endocrine Society (2017) and AAP (2023) clinical practice guidelines and is consistent with contemporary reviews of genetic obesity [1-3].

In the present patient, every effort was made to exclude these explanations: neuroimaging was normal, MC4R testing was normal, and there were no syndromic features. However, the severity and chronicity of hyperphagia suggest an imbalance in homeostatic mechanisms regulating appetite and food intake.

Impaired homeostatic and hedonic control

In normal physiology, appetite is controlled by homeostatic mechanisms (linked to energy balance) and hedonic mechanisms (linked to food reward). The arcuate nucleus serves as a node by integrating anorexigenic POMC and orexigenic neuropeptide Y (NPY) and agouti-related peptide (AgRP) signals. Dysregulation at this point, or within affiliated hedonic circuits such as the ventral tegmental area and nucleus accumbens, can result in compulsive food-seeking and perturbed satiety [4]. In our patient, the disproportional hyperphagia with intact hypothalamic imaging suggests functional imbalance within these circuits rather than structural injury.

**Figure 3:**
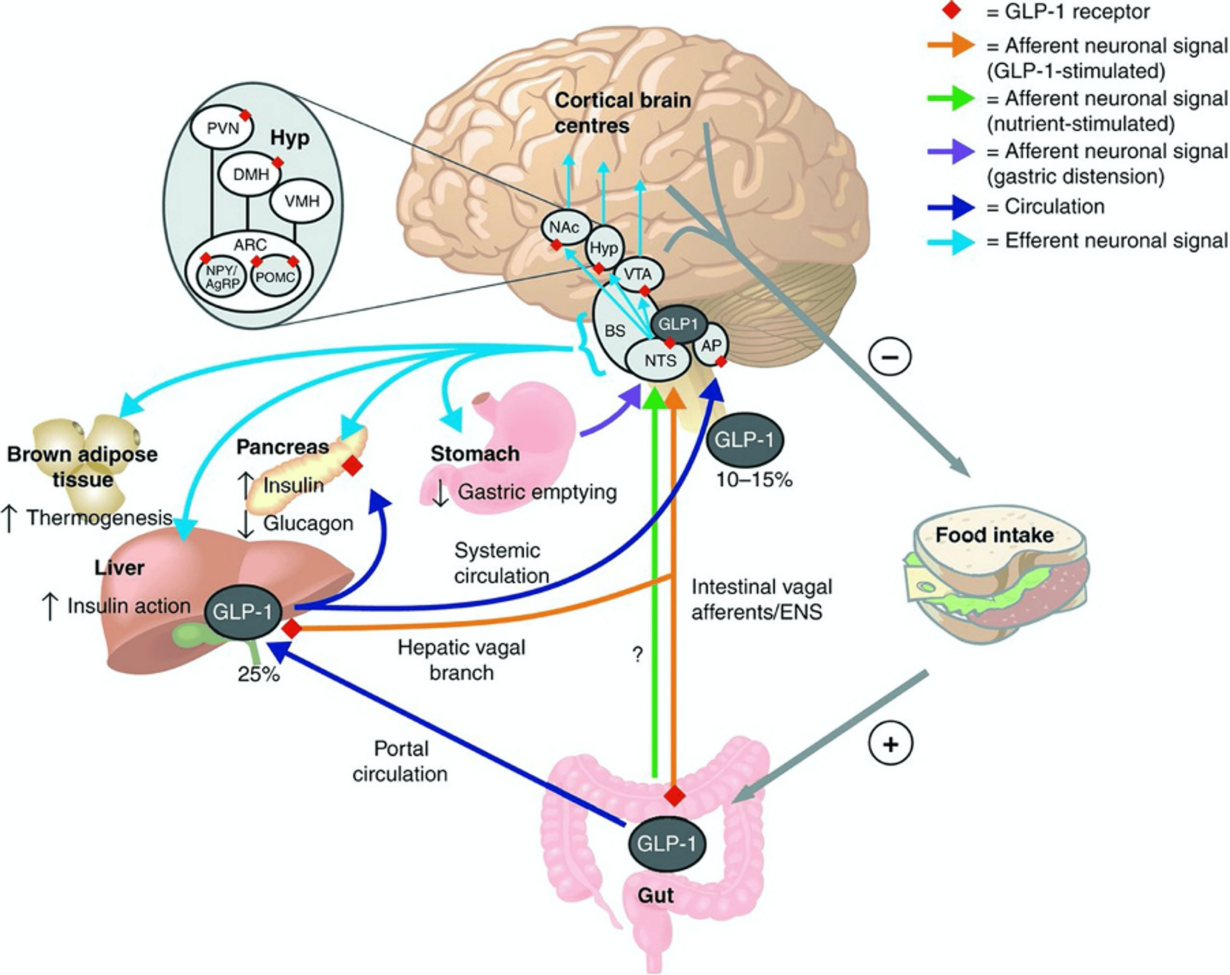
Proposed routes of action of GLP-1 in the central regulation of feeding and glucose metabolism. Adapted with permission from van Bloemendaal et al. [5], with additional concepts (including ghrelin inhibition and leptin sensitivity restoration) from Moiz et al. [4]. AgRP = agouti-related peptide; AP = area postrema; ARC = arcuate nucleus; BS = brain stem; DMH = dorsomedial hypothalamus; ENS = enteric nervous system; Hyp = hypothalamus; NAc = nucleus accumbens; NPY = neuropeptide Y; NTS = nucleus tractus solitarii; POMC = pro-opiomelanocortin; PVN = paraventricular nucleus; VMH = ventromedial hypothalamus; VTA = ventral tegmental area.

Furthermore, regions like the paraventricular nucleus (PVN) and lateral hypothalamus play an important role in the control of appetite by neuropeptides like corticotropin-releasing hormone (CRH), orexin, and thyrotropin-releasing hormone (TRH). Dysregulation in these pathways has been described in hypothalamic damage, but may also have a functional role in cases of acute early-onset obesity without identifiable structural lesions (Figure 3) [5].

Therapeutic role of tirzepatide

Tirzepatide is a 39-amino acid weekly unimolecular dual GLP-1 and GIP receptor agonist with a C20 fatty acid diacid moiety modification to prolong its half-life [6]. Through simultaneous activation of these two complementary pathways, tirzepatide more potently enhances insulin secretion, glycaemic control, and satiety than with GLP-1 therapies alone [7]. The response explains how incretin-based therapy can re-instate responsiveness to peripheral satiety signals and regulate hedonic food drive. Tirzepatide exerts its central effects through multiple complementary mechanisms. By activating GLP-1 receptors in the nucleus tractus solitarius (NTS) and area postrema of the brainstem, it enhances satiety via serotonergic and glutamatergic signaling. At the same time, suppression of dopaminergic reward pathways within the ventral tegmental area and nucleus accumbens reduces the hedonic drive to eat. In the hypothalamus, Tirzepatide promotes anorexigenic signaling by enhancing POMC activity while inhibiting NPY and AgRP pathways in the arcuate nucleus. Additional peripheral mechanisms reinforce these effects, including delayed gastric emptying, inhibition of ghrelin (hunger hormone) secretion, and restoration of leptin sensitivity, collectively leading to enhanced satiety and reduced food intake. Notably, following the initiation of tirzepatide, the patient reported a remarkable decrease in compulsive eating, and a new feeling of satiety, not previously achieved with either pharmacologic or lifestyle interventions. He remains on tirzepatide 15 mg once weekly with sustained benefit; given previous treatment refractoriness, ongoing therapy will be reviewed periodically under annual endocrinology follow-up.

Other novel multi-incretin medications, such as the triple receptor agonist retatrutide (GIP, GLP-1 and glucagon receptors), will hopefully increase available treatment options for similar cases of refractory obesity [8,9].

## Conclusions

In this adolescent with severe early-onset obesity, hyperphagia, and type 2 diabetes mellitus refractory to conventional measures, tirzepatide was associated with clinically meaningful weight loss, improved satiety, and better glycaemic indices. This case highlights the potential role of tirzepatide, a dual incretin (GLP-1/GIP) agonist, as a therapeutic option for carefully selected adolescents when standard interventions fail and has important implications for paediatric clinical decision-making. Nevertheless, given the single-patient design and short follow-up, these observations should be interpreted cautiously; longer-term, controlled paediatric studies are needed to establish efficacy, safety, and durability.
